# Characterization of Durum-Wheat Pasta Containing Resistant Starch from Debranched Waxy Rice Starch

**DOI:** 10.3390/foods12020327

**Published:** 2023-01-10

**Authors:** Mariasole Cervini, Mario Gabrielli, Giorgia Spigno, Gianluca Giuberti

**Affiliations:** Department for Sustainable Food Process (DiSTAS), Università Cattolica del Sacro Cuore, Via Emilia Parmense 84, 29122 Piacenza, Italy

**Keywords:** starch, in vitro digestion, dietary fibre, semolina, debranching

## Abstract

Durum wheat spaghetti samples prepared with increasing levels of resistant starch (RS) from debranched waxy rice starch (DWRS; i.e., 0, 5, 10, 15 g/100 g *w*/*w*) were analyzed for chemical composition, quality and sensory parameters and in vitro starch digestion. All the DWRS-containing spaghetti was “high in fibre”, the dietary fiber content being > 6 g/100 g. In addition, spaghetti with the highest level of DWRS showed the highest RS content (*p* < 0.05), being 11.4 g/100 g dry matter. The starch hydrolysis index decreased (*p* < 0.05) as the level of DWRS increased, with a reduction of >20% comparing the 15-DWRS pasta to the control. DWRS had a negative impact on quality parameters, especially at higher DWRS levels. The use of DWRS shortened the optimal cooking time and impacted the samples’ cooking loss, firmness, and stickiness. In addition, sensory analysis revealed differences among samples. However, irrespective of the level of DWRS in the recipe, the score for all attributes was > 5, which is considered the limit of acceptability. Substituting part of the semolina flour with DWRS increased the level of RS and the overall nutritional profile and affected the quality of semolina pasta, mainly at higher levels in the recipe.

## 1. Introduction

Durum wheat (i.e., *Triticum durum* Desf.) pasta is one of the most popular staple foods worldwide, with an annual production of more than 14.0 million tons [1,2]. For conventional dry pasta, durum wheat semolina is considered the best raw material due to its distinctive yellow colour, high protein and gluten content, and unique technological characteristics [2]. However, the high starch and the relatively low content in dietary fiber have stimulated research and industry to look for unconventional raw materials to include in conventional pasta products [1,2,3,4,5]. In this context, different ingredients containing bioactive compounds, dietary fibre and protein have been tested in wheat semolina pasta formulations [3,4,5,6], with resistant starch (RS) gaining attention as a replacement for digestible starch [5,7,8,9,10,11].

Dietary starch can be categorized into rapidly digestible starch (RDS), slowly digestible starch (SDS), and RS based on the rate and extent of in vitro digestion [12]. In particular, RS is a starch fraction resistant to enzymatic hydrolysis in the small intestine but, for the most part, fermented in the large intestine, acting as a substrate for microbial fermentation with dietary fibre components [12,13]. RS is desirable for the human diet because of its prebiotic effect and promising properties in reducing risk factors linked to metabolic diseases [8,13]. In addition, RS meets the definition of the Codex Alimentarius Commission for dietary fiber [12]. A greater amount of RS in pasta may reduce in vitro starch digestibility and enhance its nutritional profile, even if differences among studies exist as a function of the type of RS used [5,9,10,11,14]. This is related to the fact that certain forms of RS are not stable during heat treatment, thus reducing the total RS content in foods [11,13,15]. Consequently, research on the creation of heat-stable forms of RS, and their use in wheat-based pasta-making, is increasing.

The formation of heat-stable RS can occur with different treatments of starch [13,16,17,18]. To this end, interesting results were reported by hydrolyzing waxy rice starch (WRS) with debranching enzymes [19]. Debranching is performed by treating a starch slurry with isoamylase or pullulanase, leading to the formation of linear short-chain polymers from debranched amylopectin [20]. Studies have confirmed that debranched starch can be a good source of RS due to the realignment upon cooling of short linear chains, which can form perfect crystalline structures and expand crystalline regions that enhance the RS formation [20]. Accordingly, the use of debranched waxy rice starch (DWRS) up to 45 g/100 g *w*/*w* in gluten-free cookies contributed to formulating products with an RS content > 13 g/100 g dry matter (DM), and with marginal changes in quality attributes [21]. However, information on the functionality of DWRS in conventional wheat semolina pasta is missing.

To investigate the use of DWRS, wheat-based spaghetti samples were formulated by replacing semolina flour with increasing levels (from 0 to 15 g/100 g *w*/*w*) of DWRS. The RS content (before and after cooking) and the in vitro starch digestibility were measured. In addition, quality and sensory analyses were included to explore the role of DWRS in modifying the selected attributes.

## 2. Materials and Methods

### 2.1. Raw Materials and Ingredient Preparation

Durum wheat semolina was supplied from Barilla S.p.A. (Parma, Italy). According to the label, the chemical composition was moisture 10.7 g/100 g; crude lipid 1.5 g/100 g; total starch 71.3 g/100 g; total sugar 0.5 g/100 g; crude protein 13.5 g/100 g, and total dietary fibre 3.0 g/100 g of product. Native WRS (1.0–2.0 g amylose/100 g starch) was supplied from Riso Scotti S.p.A. (Pavia, Italy). The chemical composition was moisture 9.5 g/100 g; crude lipid 0.5 g/100 g; total starch 87.0 g/100 g; total sugar 0.2 g/100 g; crude protein 0.5 g/100 g, and total dietary fibre 0.5 g/100 g of product.

For the debranching treatment, the protocol detailed by Shi and Gao [19] was used. A WRS slurry (10% *w*/*w* in a pH 4.5 buffer solution containing 0.2 M sodium acetate and 0.2 M acetic acid) was treated at 95 °C for 30 min and then cooled to 58 °C. Afterwards, 55 ASPU/g dry starch of a heat-stable pullulanase enzyme (Diazyme^®®^ P10, 1000 ASPU/g, 1.15 g/mL; Danisco USA Inc, Beloit, WI, USA) was added to the slurry which was then kept at 58 °C under gentle agitation. After 12 h of incubation, the solution was heated at 100 °C for 25 min to inactivate the enzyme and then stored at 35 °C for 24 h. The precipitated DWRS residue, following centrifugation, was oven dried at 40 °C to a moisture content < 10 g/100 g and ground (0.5-mm screen; Lab Mill 3100; PerkinElmer Inc., Waltham, MA, USA).

### 2.2. Pasta Preparation

Durum wheat semolina was replaced with 0, 5, 10 and 15 g/100 g *w*/*w* of DWRS to obtain control (CTR), 5-DWRS, 10-DWRS and 15-DWRS pasta samples, respectively. A RZ50 pasta machine was used (La Parmigiana, Fidenza, Italy). Flour blends and tap water at 37 °C were mixed by horizontal movement (12 min) to obtain a uniform dough with a total moisture content of 34 g/100 g. A single-screw extruder with a 1.7 mm bronze spaghetti-shaped die was used. The temperature was kept < 50 °C, and the auger screw extrusion speed was 20 rpm. Spaghetti samples were cut at 20 cm in length and dried at 50 °C for 11 h (La Parmigiana ESS20). For each recipe, three batches were produced.

### 2.3. Chemical Composition

The chemical composition of raw pasta samples was assessed using AOAC official methods of analysis [22]. Total starch was evaluated using the AOAC method 996.11 [22]. An enzyme assay kit (Megazyme K-INTDF 02/15) was used for the measurement of the total dietary fibre content (TDF). The RS content in flours (i.e., DRWS and semolina), and in uncooked and cooked spaghetti samples was assessed enzymatically (K-RSTAR 02/17, Megazyme International, Wicklow, Ireland) [22,23,24].

### 2.4. Colour Evaluation

The surface colour of uncooked and cooked samples (CIELAB system colour space; L*, a*, b*) was assessed using a Minolta CR410 Chroma Meter (Konica Minolta Co., Tokyo, Japan; D65 standard illuminant; visual angle of 10). Seven readings were taken for each sample. The total colour difference (ΔE*) was calculated using the following formula:

ΔE*_s-ctr_ = [(L*_s_ − L*_ctr_)^2^ + (a*_s_ − a*_ctr_)^2^ + (b*_s_ − b*_ctr_)^2^]^1/2^ (1)

where: s = DWRS-spaghetti and ctr = control spaghetti. A ΔE* value greater than 3 was used to verify whether the colour differences were visible to the human eye [23].

### 2.5. Thermal Properties, Pasta Quality, and Texture

The thermal properties of raw samples were assessed using differential scanning calorimetry (DSC Setline^®^, Setaram, Denmark) following the procedure and sample preparation detailed by Cervini et al. [24]. The onset temperature (T_o_), the peak temperature (T_p_), the conclusion temperature (T_c_) and the gelatinization enthalpy (ΔH) were recorded.

The optimal cooking time (OCT), the cooking loss (CL) and the water absorption capacity (WAC) were determined with the AACC approved method 66-50 [25].

The firmness and stickiness values of cooked samples (AACC method 66-50) were measured with a TA-XT2i Texture Analyzer (Stable Micro Systems, Godalming, UK) with a 5 kg load cell [24,25]. For texture analysis, 15 strands of cooked spaghetti were aligned over a stainless-steel platform. A light knife blade (A/LKB) was used to assess firmness, whereas a firmness/stickiness rig (HDP/PFS) was used for stickiness evaluation [25]. For firmness, a speed of 0.17 mm/s was employed, whereas for stickiness a compression speed of 0.5 mm/s and a compression force of 1 kg for 2 sec were used [25]. Ten measurements for each sample were made.

### 2.6. In Vitro Starch Digestion of Cooked Pasta

After a simulated mastication step using a meat mincer, cooked samples (i.e., 2 g) were subjected to a gastric digestion phase in a 0.05 M HCl solution (pH = 2) containing pepsin (5 mg/mL; Sigma P 7000; Sigma-Aldrich Co., Milan, Italy) for 30 min at 37 °C under agitation [24]. Then, the pH was adjusted to 5.2 by adding 0.1 M sodium acetate buffer, and the pancreatic phase at 37 °C was simulated through the addition of pancreatin (7500 FIP-U/g; Merck 7130, Merck KGaA, Darmstadt, Germany), amyloglucosidase (300 U/mL; Sigma A-7095, Sigma-Aldrich Co., Milan, Italy) and invertase (300 U/g; Sigma I-4504, Sigma-Aldrich Co., Milan, Italy) [24,26]. Aliquots (2 mL) were taken every 30 min up to 180 min of the pancreatic phase, mixed with absolute ethanol, and the amount of the released glucose was determined (GODPOD 4058, Giesse Diagnostic snc, Rome, Italy). The percentage of digested starch was calculated using a factor of 0.9. The starch hydrolysis index (HI) was derived from the area under the starch hydrolysis curve (0–180 min) with white wheat bread (total starch content of 72.3 g/100 g DM), as a reference [24,26]. Analyses were run in triplicate.

### 2.7. Sensory Analysis

The sensory attributes of spaghetti were assessed by a voluntary 47-member panel composed of students and staff of the Department for Sustainable Food Process (DiSTAS), (43% males and 57% females, 20–51 years old). Each panelist received 6 h of training and completed a written informed consent. A three-digit random code was used for sample identification [24]. Colour uniformity, appearance, texture, aroma, taste, and springiness were evaluated. A 9-point hedonic scale was used for assigning the intensity of liking and disliking. Overall acceptability was assessed using the same hedonic scale. For all attributes, a score ≥ 5 was considered the limit of acceptability [27].

### 2.8. Statistical Analyses

Data are presented as the mean values ± standard deviation of at least three replicates. The analysis of variance (One-way ANOVA) with a post hoc Tukey test at *p* < 0.05 using IMB SPSS Statistics software (Version 25) was used for data comparison.

## 3. Results

### 3.1. Chemical Composition and Resistant Starch Content

Irrespective of the DWRS level, durum wheat-based spaghetti had similar humidity and crude lipid contents, with average values of 10.3 g water/100 g and 1.2 g/100 g DM, respectively (Table 1). As expected, a decrease (*p* < 0.05) in the total starch and crude protein contents was measured with increasing levels of DWRS. Similar results have been reported in gluten-free pasta containing increasing amounts of a RS ingredient from annealed sorghum starch [24]. The TDF content increased in line with the level DWRS, the highest value recorded for 15-DWRS (i.e., 12.9 g/100 g DM, *p* < 0.05). Moreover, all the DWRS-containing samples were “high in fibre”, the dietary fiber content being >6 g/100 g [1,8]. It should be noted that the analytical procedure used in assessing the TDF includes the RS and the non-digestible oligosaccharide contents in the calculation of this food component [28,29,30].

In this study, the RS content of spaghetti was measured before and after the cooking step to verify the thermal stability of the DWRS. The consumption of RS has been characterized by promising effects on human health, including improved glucose tolerance, greater cellular sensitivity to insulin, and increased post-meal satiety [31,32]. However, the native RS content can be reduced during starch gelatinization by disrupting the semicrystalline structure of the starch granules [13,14,24]. Accordingly, Gelencsér et al. [17] formulating pasta by replacing 20% (*w*/*w*) of durum wheat with two different RS-rich ingredients, reporting an RS loss after cooking of about 50%. By contrast, Aravind et al. [9] did not report changes in the RS content of durum wheat pasta formulated with two different RS ingredients before and after the cooking step.

Concerning the raw ingredients, the RS content of the DWRS was 68.8 g/100 g DM, thus confirming the suitability of debranching WRS to produce RS [19]. In addition, the RS of wheat semolina was 2.3 g/100 g DM. Uncooked control pasta was characterized by an RS content of 1.9 g/100 g DM, in line with previous findings [9,11]. After cooking, the RS content of CTR decreased to 0.2 g/100 g DM, with a calculated RS loss value > 90%. The substitution of semolina flour with the DWRS ingredient increased the RS content of spaghetti. In particular, the highest (*p* < 0.05) RS content was measured in 15-DWRS spaghetti, being 11.4 g/100 g DM in the raw form, and 9.3 g/100 g DM after cooking. Considering the RS content of DWRS-spaghetti prior and after cooking, an average estimated RS loss values < 20% was calculated, irrespective of the substitution level. Treating WRS with pullulanase can create more ordered crystalline structures with adequate heat stability to maintain their close packing under cooking [20]. During debranching, starches can release short linear chain glucans from amylopectin that can easily re-associate, leading to a tight crystalline solid structure which can resist disruption during gelatinization [33,34,35]. Current findings agreed with those reported in gluten-free cookies formulated with 50% of DWRS, even if lower heat stability values were reported (about 50%) [21]. Considering heat stability, the selected RS-rich ingredient (i.e., DWRS) appears more suitable to be used in pasta than in baked food production.

### 3.2. Colour of Pasta Samples before and after Cooking

Colour changes before and after cooking are presented in Table 2. In general, samples containing DWRS had higher lightness (i.e., L*) and lower yellowness (i.e., b*) values than the control (*p* < 0.05), irrespective of the cooking process and the level of substitution. This was related to the whiteness of the DWRS ingredient. Aravind et al. [9] showed a decrease in b* values of wheat-based pasta formulated with increasing amounts of RS, and an increase in L* values was reported in durum wheat spaghetti formulated with different levels of a RS-rich ingredient from phosphorylated cross-linked wheat starch [36]. In addition, different ΔE* values were measured (*p* < 0.05). Prior to and after cooking, the 10-DWRS and 15-DWRS samples were characterized by ΔE* values > 3 with respect to the control, meaning that the human eye can distinguish these samples.

### 3.3. Thermal Properties and Pasta Quality

The T_o_, T_p_, T_c_, and ΔH mean values of CTR pasta were 60.3 °C, 68.2 °C, 74.4 °C and 6.2 J/g dry starch, respectively (Table 3). Namir et al. [37] reported that 100% wheat-based extruded pasta had a thermal transition peak of 59–67 °C, in line with present findings.

All DWRS-spaghetti had well-defined transition temperatures (T_o_, T_p_, and T_c_). By increasing the substitution level of semolina flour with DWRS, the gelatinization transition temperatures increased (*p* < 0.05) from 61.9 to 67.2 °C for T_o_, from 67.5 to 72.4 °C for T_p_, and from 73.4 to 77.2 °C for T_c_, indicating greater thermal stability of semolina pasta added with DWRS. Both 10-DWRS and 15-DWRS pasta required more energy to gelatinize starch (mean value of 9.0 J/g dry starch) than the other samples. These difference in the starch gelatinization properties appear consistent with the inherent level of DWRS in the recipe. Accordingly, previous studies indicated that the increase in the RS content could lead to higher ΔH values, suggesting a formation of stable double helical structures following linear short-chain alignment and aggregation [20,33]. In addition, higher gelatinization temperatures can indicate a more rigid internal crystalline structure of starch with greater heat stability [34]. Accordingly, Cao et al. [38] and Wang et al. [35] reported a shift toward higher thermal transition temperatures for debranched rice starch than native starch.

The OCT was reduced in the RS-incorporated spaghetti, varying from 11.2 min for CTR to 9.5 min for 15-DWRS (*p* < 0.05). Aravind et al. [9] reported shorter OCT in semolina pasta formulated with different blends of wheat semolina:RS-rich ingredients. In particular, the authors reported a reduction in the OCT of about 12% comparing control semolina pasta to RS-containing samples.

CL is an indicator of the capacity of the gluten-starch network to retain its physical integrity during cooking. In general, in durum wheat pasta, CL values should not exceed the threshold value of 8% [11]. As reported in Table 3, both 10-DWRS and 15-DWRS pasta were characterized by higher CL values (*p* < 0.05) than in the other samples. It has been reported that adding any other ingredient not part of the wheat or gluten can contribute to diluting gluten strength, thus allowing more solids to be released into the cooking water [11]. Similar results were reported by Sozer et al. [39] in pasta enriched with 10% of RS, and by Aravind et al. [9] with a level of inclusion of RS > 10%.

Lower WAC values were measured as the level of DWRS increased (*p* < 0.05). A shorter cooking time can correspond to decreased water absorption due to less starch granule hydration [39]. Aravind et al. [9] showed that pasta WAC decreased with RS substitution levels > 10%, whereas Gelencsér et al. [17] found no difference in WAC in RS-containing pasta at 10% and 20% substitution. The different characteristics of the RS-rich ingredients used among studies may explain these discrepancies.

Firmness and stickiness are critical parameters used for the cooking quality of pasta. As reported in Table 2, the addition of DWRS significantly decreased the firmness (as maximum cutting force) of the cooked pasta, ranging from 2.8 N to 1.7 N for control and 15-DWRS spaghetti, respectively (*p* < 0.05). Firmness in pasta is highly correlated with gluten content [9]. A decline in firmness following increasing substitution levels of semolina with non-gluten flours could be expected [9]. In addition, the decrease in pasta firmness associated with the increase of DWRS in the formulation agrees with the increase in CL values.

High-quality cooked pasta should have minimal stickiness values [32]. As reported in Table 2, stickiness values increased as the level of DWRS was > 10 g/100 g *w*/*w* in the recipe, the highest value recorded for 15-DWRS (i.e., 2.0 N: *p* < 0.05). The stickiness of semolina pasta results from constituents escaping from the protein network, mainly amylopectin, and adhering to the surface of cooked pasta [4,40]. In this case, following DWRS inclusion, pasta became stickier, probably due to the weakening of the gluten network and starch granules less incorporated in a protein matrix [40].

### 3.4. In Vitro Starch Digestion of Cooked Pasta

**Figure 1:**
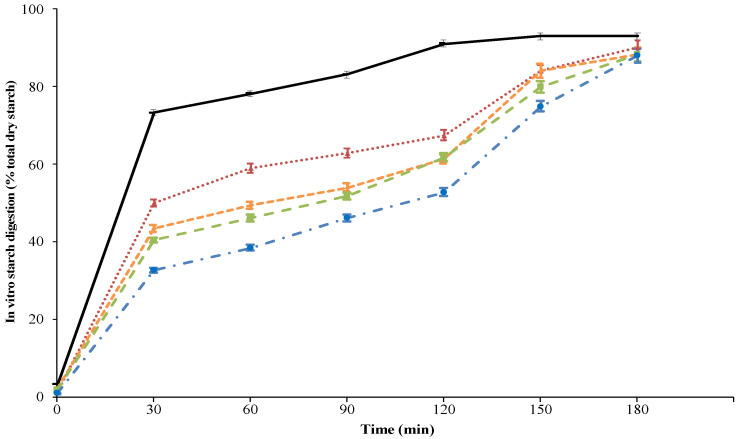
In vitro starch digestion of semolina spaghetti with resistant starch from debranched waxy rice starch (DWRS). Control: spaghetti with 100% *w*/*w* durum wheat flour (red line); 5-DWRS: spaghetti with durum wheat flour and DWRS 95:5 *w*/*w* (orange line); 10-DWRS: spaghetti with durum wheat flour and DWRS 90:10 *w*/*w* (green line); 15-DWRS: spaghetti with durum wheat flour and DWRS 85:15 *w*/*w* (blue line). White wheat bread was used as reference (black line).

Compared to spaghetti, the extent of the in vitro starch digestion of starch from white wheat bread was greater for the entire incubation period (Figure 1), in line with previous findings [24]. The different RS content of samples (i.e., control, 5-DWRS, 10-DWRS and 15-DWRS) were reflected in the different extent of in vitro starch digestion (Figure 1). Consequently, the starch HI decreased (*p* < 0.05) as the level of DWRS increased in the formulation, ranging from 76.1 to 61.9 for control and 15-DRWS, respectively. The lower accessibility of DWRS to enzymatic digestion can contribute to explaining present findings, as already reported in gluten-free rice-based cookies [21].

The lower enzyme accessibility of DWRS can be attributed to recrystallization of the linear short-chain molecules from debranched amylopectin during retrogradation, and to compact double helical structures through hydrogen bonds [19]. In particular, the debranching treatment generates free branches which can act like amylose and can create highly crystalline structures with limited enzyme accessibility [18,20,21]. In addition, the RS fraction does not contribute to the release of glucose during enzyme hydrolysis, thus reducing the starch HI of selected starch-based food products [32,41]. Comparable results were obtained for gluten-free pasta incorporating an RS ingredient from annealed sorghum starch, and in semolina spaghetti formulated with two different RS-rich ingredients at comparable inclusion levels [9,21]. Lastly, the lower starch content in spaghetti because of DWRS inclusion, and the different behavior of the starch system related to possible interactions among wheat semolina and DWRS following extrusion and cooking, could further explain our in vitro findings. For instance, greater RS contents can influence the digestibility of the available starch fraction by the encapsulation of gelatinized starch between layers of RS [23,24,32]. However, to confirm the present in vitro findings, in vivo trials are strongly recommended.

### 3.5. Sensory Analysis

None of the DWRS-containing pasta was significantly different in aroma and taste attributes from the control, with average values of 6.9 and 7.0, respectively (Table 4). This is related to the neutral flavour of the DWRS ingredient [21,22,23,24,25,26,27,28,29,30,31,32,33,34,35,36,37,38,39,40,41]. Similarly, Gelencsér et al. [17] indicated similar aroma and taste values between wheat pasta enriched with RS compared to the control. In addition, the sensory scores for colour and texture between control and DWRS-containing pasta appeared consistent with the instrumental values (Table 2). In particular, the lowest sensory scores for colour and texture were obtained for 15-DWRS spaghetti (i.e., 5.4 and 5.5, respectively; *p* < 0.05), confirming the impact of DWRS in modifying the sensory attributes. This contrasts with data presented by Aravind et al. [9], where minimal differences were detected for the sensory attributes comparing RS-enriched wheat pasta to the control.

The overall acceptance decreased as the level of DWRS increased in the formulation, ranging from 8.2 to 6.4 for CTR and 15-DWRS pasta, respectively (*p* < 0.05). Similarly, Cervini et al. [24] reported a decrease in the overall acceptance of gluten-free rice pasta formulated with increasing levels of RS obtained from annealed sorghum starch. Bustos et al. [42] obtained similar results by comparing the overall acceptability of wheat-based pasta to RS-enriched counterparts. However, it should be noted that, despite the reported decrease in the overall acceptance, all samples resulted in a higher than 5, which is considered the limit of acceptability [27].

## 4. Conclusions

The current work explored the use of DWRS in wheat-based pasta formulation by exploring the nutritional, quality, and sensory attributes of newly developed products. The substitution of semolina flour starting from 5 g/100 g *w*/*w* of DWRS increased the RS content while lowering the starch HI. In addition, DWRS inclusion allows using the “high in fibre” nutritional claim [43]. From a technological standpoint, the use of DWRS shortened the cooking time, increased cooking losses, and changed texture and colour values, which may have implications on the attractiveness of DWRS-containing pasta. Sensory attributes were also affected, even if the overall acceptance exceeded the limit of acceptability. Further studies are therefore required to assess in vivo potential health benefits of the novel pasta, and to optimize the level of DWRS inclusion to limit undesirable quality changes.

## Figures and Tables

**Table 1:** Chemical composition (g/100 g dry matter) of semolina spaghetti containing resistant starch (RS) from debranched waxy rice starch (DWRS).

	Pasta Samples			
	Control ^1^	5-DWRS ^2^	10-DWRS ^3^	15-DWRS ^4^
Humidity (g water/100 g)	10.4 ± 0.21 ^a^	10.2 ± 0.42 ^a^	10.6 ± 0.56 ^a^	10.2 ± 0.07 ^a^
Total starch	73.4 ± 2.37 ^a^	70.1 ± 1.45 ^b^	68.6 ± 1.98 ^c^	65.8 ± 2.10 ^d^
Crude protein	11.4 ± 0.13 ^a^	10.1 ± 0.35 ^b^	9.0 ± 0.32 ^c^	8.3 ± 0.77 ^d^
Crude lipid	1.2 ± 0.01 ^a^	1.3 ± 0.05 ^a^	1.3 ± 0.19 ^a^	1.2 ± 0.04 ^a^
Ash	0.6 ± 0.02 ^a^	0.5 ± 0.02 ^a^	0.4 ± 0.05 ^a^	0.3 ± 0.02 ^b^
Total dietary fiber	2.4 ± 0.27 ^d^	6.4 ± 0.71 ^c^	8.8 ± 0.71 ^b^	12.9 ± 1.31 ^a^
RS (uncooked sample)	1.9 ± 0.11 ^d^	5.1 ± 0.27 ^c^	7.3 ± 0.43 ^b^	11.4 ± 1.04 ^a^
RS (cooked sample)	0.2 ± 0.01 ^d^	4.3 ± 0.68 ^c^	5.9 ± 0.49 ^b^	9.3 ± 0.67 ^a^

Means in the same line with different superscripts differed at *p* < 0.05. ^1^ Spaghetti with 100% *w*/*w* durum wheat flour. ^2^ Spaghetti with durum wheat flour and DWRS 95:5 *w*/*w*. ^3^ Spaghetti with durum wheat flour and DWRS 90:10 *w*/*w*. ^4^ Spaghetti with durum wheat flour and DWRS 85:15 *w*/*w*.

**Table 2:** Colour evaluation of semolina spaghetti containing resistant starch (RS) from debranched waxy rice starch (DWRS).

	Pasta Samples			
	Control ^1^	5-DWRS ^2^	10-DWRS ^3^	15-DWRS ^4^
Uncooked				
Lightness L*	75.4 ± 0.21 ^d^	76.4 ± 1.32 ^c^	81.2 ± 0.43 ^b^	83.8 ± 1.27 ^a^
Redness a*	1.4 ± 0.32 ^b^	1.5 ± 0.01 ^b^	1.5 ± 0.12 ^b^	1.7 ± 0.18 ^a^
Yellowness b*	57.5 ± 1.15 ^a^	54.0 ± 0.84 ^b^	51.1 ± 1.73 ^c^	49.3 ± 2.01 ^d^
ΔE*	-	2.1	4.3	5.8
Cooked to optimum				
Lightness L*	71.8 ± 1.48 ^d^	73.1 ± 1.51 ^c^	76.4 ± 1.14 ^b^	79.6 ± 1.77 ^a^
Redness a*	1.1 ± 0.01 ^a^	0.9 ± 0.01 ^a^	1.0 ± 0.02 ^a^	1.1 ± 0.01 ^a^
Yellowness b*	53.4 ± 0.11 ^a^	49.3 ± 0.43 ^b^	44.9 ± 0.21 ^c^	40.4 ± 0.51 ^d^
ΔE*	-	1.6	3.9	5.2

Means in the same line with different superscripts differed at *p* < 0.05. ^1^ Spaghetti with 100% *w*/*w* durum wheat flour. ^2^ Spaghetti with durum wheat flour and DWRS 95:5 *w*/*w*. ^3^ Spaghetti with durum wheat flour and DWRS 90:10 *w*/*w*. ^4^ Spaghetti with durum wheat flour and DWRS 85:15 *w*/*w*.

**Table 3:** Thermal properties and quality parameters of semolina spaghetti containing resistant starch (RS) from debranched waxy rice starch (DWRS).

	Pasta Samples			
	Control ^1^	5-DWRS ^2^	10-DWRS ^3^	15-DWRS ^4^
Thermal properties				
Onset temperature T_o_ (°C)	59.7 ± 1.56 ^d^	61.9 ± 1.43 ^c^	66.2 ± 1.03 ^a^	67.2 ± 1.47 ^a^
Peak temperature T_p_ (°C)	64.3 ± 1.42 ^c^	67.5 ± 0.95 ^b^	71.8 ± 1.27 ^a^	72.4 ± 2.17 ^a^
Conclusion temperature T_c_ (°C)	69.5 ± 1.61 ^c^	73.4 ± 1.04 ^b^	76.2 ± 1.82 ^a^	77.2 ± 1.31 ^a^
Gelatinization enthalpy ΔH (J/g dry starch)	6.2 ± 0.98 ^c^	7.4 ± 0.91 ^b^	8.8 ± 0.61 ^a^	9.2 ± 0.75 ^a^
Pasta quality parameters				
Optimal cooking time (min)	11.2 ± 0.28 ^a^	10.7 ± 0.43 ^b^	10.1 ± 0.17 ^b^	9.5 ± 0.24 ^c^
Cooking loss (%)	5.5 ± 0.23 ^c^	5.7 ± 0.49 ^c^	6.4 ± 0.49 ^b^	7.3 ± 0.66 ^a^
Water absorption capacity (%)	154.4 ± 4.42 ^a^	149.9 ± 3.21 ^a^	137.5 ± 3.61 ^b^	119.1 ± 4.11 ^c^
Firmness (N)	2.8 ± 0.18 ^a^	2.6 ± 0.41 ^a^	2.1 ± 0.16 ^b^	1.7 ± 0.08 ^c^
Stickiness (N)	1.5 ± 0.10 ^c^	1.6 ± 0.14 ^c^	1.8 ± 0.11 ^b^	2.0 ± 0.08 ^a^
In vitro starch digestion				
Starch hydrolysis index ^5^	76.1 ± 2.13 ^a^	71.6 ± 3.01 ^b^	65.5 ± 2.31 ^c^	61.9 ± 2.01 ^d^

Means in the same line with different superscripts differed at *p* < 0.05. ^1^ Spaghetti with 100% *w*/*w* durum wheat flour. ^2^ Spaghetti with durum wheat flour and DWRS 95:5 *w*/*w*. ^3^ Spaghetti with durum wheat flour and DWRS 90:10 *w*/*w*. ^4^ Spaghetti with durum wheat flour and DWRS 85:15 *w*/*w*. ^5^ Calculated using white wheat bread as reference (starch hydrolysis index = 100).

**Table 4:** Sensory scores of semolina spaghetti containing resistant starch (RS) from debranched waxy rice starch (DWRS).

	Pasta Samples			
	Control ^1^	5-DWRS ^2^	10-DWRS ^3^	15-DWRS ^4^
Colour	7.4 ± 0.94 ^a^	6.8 ± 0.72 ^b^	6.3 ± 0.94 ^c^	5.4 ± 0.54 ^b^
Appearance	7.1 ± 0.62 ^a^	7.2 ± 0.14 ^a^	7.0 ± 0.93 ^a^	7.1 ± 0.11 ^a^
Texture	7.8 ± 0.13 ^a^	6.8 ± 0.56 ^b^	6.2 ± 0.93 ^c^	5.5 ± 0.62 ^d^
Aroma	6.9 ± 0.16 ^a^	7.0 ± 0.18 ^a^	7.1 ± 0.33 ^a^	6.9 ± 0.48 ^a^
Taste	7.0 ± 0.11 ^a^	7.1 ± 0.67 ^a^	7.1 ± 0.61 ^a^	6.9 ± 0.96 ^a^
Overall acceptance	8.2 ± 0.89 ^a^	7.6 ± 0.65 ^b^	6.7 ± 2.01 ^c^	6.4 ± 1.16 ^c^

Means in the same line with different superscripts differed at *p* < 0.05. ^1^ Spaghetti with 100% *w*/*w* durum wheat flour. ^2^ Spaghetti with durum wheat flour and DWRS 95:5 *w*/*w*. ^3^ Spaghetti prepared with durum wheat flour and DWRS 90:10 *w*/*w*. ^4^ Spaghetti with durum wheat flour and DWRS 85:15 *w*/*w*.

## Data Availability

Data are contained within the article.
